# Exploring the Siderophore Portfolio for Mass Spectrometry-Based Diagnosis of Scedosporiosis and Lomentosporiosis

**DOI:** 10.1021/acsomega.4c08257

**Published:** 2024-10-23

**Authors:** Jiří Houšt’, Andrea Palyzová, Tomáš Pluháček, Jiří Novák, Helena Marešová, Petr Hubáček, Radim Dobiáš, David A. Stevens, Hélène Guegan, Jean-Pierre Gangneux, Vladimír Havlíček

**Affiliations:** † Laboratory of Molecular Structure Characterization, Institute of Microbiology of the Czech Academy of Sciences, Vídeňská 1083, 142 00 Prague, Czechia; ‡ Department of Analytical Chemistry, Faculty of Science, Palacký University in Olomouc, 17. listopadu 1192/12, 779 00 Olomouc, Czechia; § Department of Software Engineering, Faculty of Information Technology, Czech Technical University in Prague, Thákurova 9, 160 00 Prague, Czechia; ∥ Department of the Medical Microbiology, second Faculty of Medicine, Charles University and Motol University Hospital, V Úvalu 84, 150 06 Prague, Czechia; ⊥ Department of Bacteriology and Mycology, National Reference Laboratory for Mycological Diagnostics, Public Health Institute in Ostrava, Partyzánské náměstí 2633/7, 702 00 Ostrava, Czechia; # Institute of Laboratory Medicine, Faculty of Medicine, University of Ostrava, Syllabova 19, 703 00 Ostrava, Czechia; ¶ Division of Infectious Diseases and Geographic Medicine, Stanford University School of Medicine, Foundation for Research in Infectious Diseases, P.O. Box 2734, Saratoga, California 95070, United States; ∇ Division of Parasitology and Mycology, European Excellence Center in Medical Mycology (ECMM EC), National Reference Center on Chronic Aspergillosis, Rennes University Hospital, Inserm UMR_S 1085 Irset, 2 Rue Henri le Guilloux, 35033 Rennes, France

## Abstract

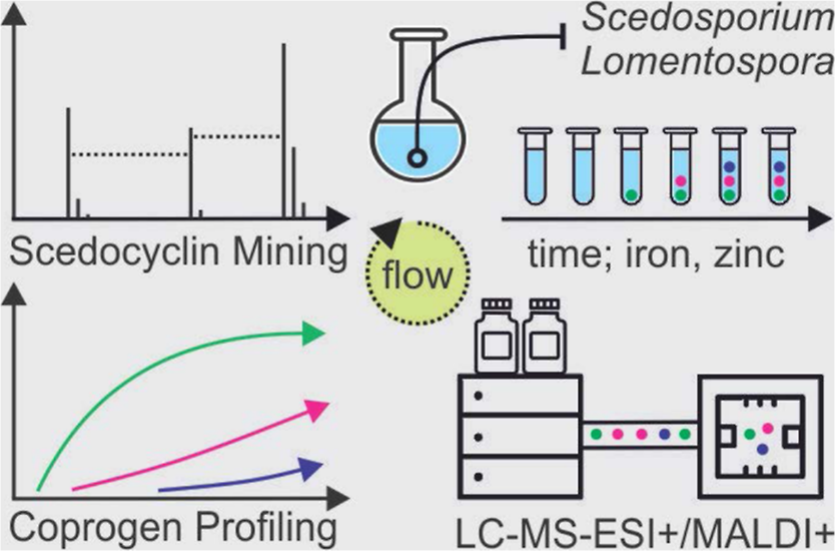

*Scedosporium apiospermum* and *Lomentospora prolificans* secrete siderophores (iron scavengers) during hyphal proliferation. Siderophores are virulence factors and potential clinical biomarkers of invasive scedosporiosis and lomentosporiosis. Both strains secreted a uniform spectrum of siderophores, including coprogen B (CopB), *N*^α^-methyl-coprogen B, dimethyl-coprogen, and ferricrocin, with *N*^α^-methyl-coprogen B being the fastest secreted and most abundant coprogen. Under iron and zinc restriction, reflecting a nutrient-limited host environment, *L. prolificans* secreted 45 times more CopB than did *S. apiospermum*, presumably contributing to its higher virulence. This robust mobilization of CopB was further enhanced by zinc surplus. Additionally, two novel cyclic peptides, Scedocyclin A and B, were characterized in*Scedosporium boydii* using the *de novo* sequencing tool CycloBranch. Utilizing matrix-assisted laser desorption/ionization, the portfolio of coprogens detected had limits of detection and quantitation of 4.9 and 14.6 fmol/spot in complex matrices, respectively, making them strong candidates for the next-generation, routine diagnosis of invasive scedosporiosis and lomentosporiosis through the Biotyper siderotyping.

## Introduction

*Scedosporium* species and *Lomentospora prolificans* are saprophytic filamentous fungi listed on the World Health Organization’s list of priority fungal pathogens for humans.^1^ They are gaining worldwide attention as increasingly isolated opportunistic fungal pathogens intrinsically resistant to clinically available antifungals.^2−4^ Ubiquitously distributed in human environments, direct contact with their conidia, whether aspirated or traumatically inoculated, may result in diverse forms of scedosporiosis or lomentosporiosis, depending on the current state of host immunity and their virulence factors such as melanin.^5,6^ Immunocompetent patients often develop localized, cutaneous or soft tissue eumycetoma^7^ or are asymptomatically colonized in pre-existing pulmonary cavities. *Scedosporium* and *Lomentospora* are not uncommonly found in the lungs of cystic fibrosis (CF) patients, which may subsequently proceed to invasive mycoses.^8^ In contrast, immunocompromised patients, such as those with leukemia, lymphoma, or transplantation, experience invasive pulmonary or disseminated scedosporiosis or lomentosporiosis with a mortality rate approaching 90%.^9^ The predominant strains that cause life-threatening infections are*Scedosporium apiospermum*(*Sa*),*Scedosporium boydii*(*Sb*), and *L. prolificans* (*Lp*).^10^

Microbiological diagnosis of *Scedosporium* and *Lomentospora* requires preferentially semiselective cultivation media for their effective isolation from nonsterile anatomical sites, often contaminated with faster-growing *Aspergillus fumigatus* (*Af*).^11^ Gene analysis of the β-tubulin, calmodulin, and internal transcribed spacer region of the rRNA gene is used for interspecies identification within the *Sa* complex (*S. apiospermumsensu stricto*, *Scedosporium aurantiacum*, *Scedosporium dehoogii*, *S. boydii*, and *Scedosporium minutispora*).^12^ In addition, not-yet-standardized enzyme-linked immunosorbent assays were developed for the serodiagnosis of *Sb* catalase A1 and Cu/Zn-superoxide dismutase,^13^ as well as for the specific anti-*Sb* immunoglobulin G.^14^ Both assays have shown a minimum of 86% sensitivity and 93% specificity compared to the presumptive diagnostics based on detecting pan-fungal 1,3-β-d-glucans.^15^

In clinically important fungi, secondary metabolites are recognized as virulence factors and can offer a deeper look inside proliferating cells with active secondary metabolism, as opposed to circulating fragments of dead fungal bodies or other products.^16,17^ Among others, fungal siderophores are gaining more attention as promising biomarkers of invasive mycoses.^18^ Siderophores are a subclass of metallophores that are biosynthesized under iron-limited conditions, including in a host, to scavenge ferric ions due to the increased metabolic activity needs of germinating conidia and proliferating hyphae.^19^ As an example, the major extracellular *Af* siderophore triacetylfusarinine C (TafC) was successfully recovered (sensitivity 92%, specificity 100%) from the urine of 13 patients diagnosed with probable invasive pulmonary aspergillosis,^20^ using an infection metallomics-based approach.^21^ Concurrently, another extracellular siderophore, *N*^α^-methyl-coprogen B (*N*-CopB), was detected in vitro^22^ and in sputum samples, correlating with a respiratory infection site in CF patients, whose sputum also grew *Sa*.^23^ Thus, far, there has been no report of clinical diagnostic application of coprogens in fluids distant from the infection site, such as urine.

Four iron acquisition mechanisms have been described in fungi: low-affinity uptake of ferrous ions via divalent cation carriers, high-affinity reductive iron assimilation, siderophore-mediated iron acquisition (SIA), and heme uptake and degradation.^24^ So far, the most advanced characterization of iron acquisition, homeostasis, and metabolism was described for *Af*.^19^ Analogous to studies of *Af*, studies in *Sa* conducted by Le Govic et al.^25,26^ found orthologous genes involved in siderophore biosynthesis (both extracellular and intracellular), transport, and regulation, indicating the significance of SIA in *Sa* propagation. Indeed, disrupting the *sidD* gene, which drives the biosynthesis of *Af* extracellular siderophores, resulted in attenuated virulence of *Sa* in a murine model of disseminated scedosporiosis and abolished the biosynthesis of *N*-CopB in a parallel *in vitro* study.^27^ This indicates that the genome of *Sa* contains biosynthetic machinery comparable to *Af* for producing extracellular siderophores. In *Sa*, no reports address intracellular siderophores such as ferricrocin (Fc). While the whole genome sequence of *Lp* was published in 2017,^28^ no reports regarding genes and siderophores involved in SIA are currently available.

Similarly, zinc regulation of SIA has not been explored in both *Scedosporium* and *Lomentospora*. In *Af*, two crucial iron-sensing regulators—zinc-inducible SreA and basic leucine zipper HapX—are indispensable for the transcription of genes involved in iron homeostasis.^29^ Under an iron surplus, SreA represses HapX and the siderophore system to prevent iron toxicity. Conversely, HapX suppresses SreA and iron-consuming pathways while activating siderophore biosynthesis during iron starvation.^19^ Both SreA and HapX are highly conserved in the fungal phyla Ascomycota (including *Af*, *Sa*, and *Lp*), Basidiomycota, and Mucoromycota.^29^ Besides SreA, zinc cluster transcription factor AcuM and Zn_2_Cys_6_ transcription factor AtrR are required to produce the *Af* extracellular siderophores during iron starvation. Deletion of these factors led to a reduction in *hapX* gene transcript levels, resulting in the decreased content of extracellular siderophores by 80% and 20%, respectively.^30,31^ Additionally, the deletion of AtrR decreased the Fc content by 14%.^31^

Limited information has been provided on the metabolome of *Sa* and *Lp*, specifically regarding extracellular^27^ and intracellular siderophore secretion.^3^ For *Sb*, originally described as *Pseudallescheria boydii*,^32^ the list of fungal secondary metabolites has not been updated since 2011.^33^ In our study, we report differences in the secretion kinetics of siderophores between *Sa* and *Lp* strains affected by zinc and iron stresses and identify key coprogens whose overproduction could reflect the rapidly increasing proliferation and the pathogen virulence under different stress conditions. The straightforward invasive scedosporiosis and lomentosporiosis diagnosis by Biotyper siderotyping is proposed, followed by characterization of two novel scedocyclins associated with *Sb*.

## Materials and Methods

### Cultivation of *S. apiospermum* and *L. prolificansSensu Stricto*

*Sa* (strain M1643) and *Lp* (strain MY21539) were fungal isolates obtained from the Public Health Institute (Ostrava, Czechia) and Motol University Hospital (Prague, Czechia), respectively. For the siderophore profiling, the strains were grown (37 °C, 1 week) on Sabouraud Dextrose Agar (Oxoid, Basingstoke, UK). Conidia were harvested using phosphate-buffered saline containing 0.1% Tween 80 and inoculated (10^5^ spores/mL) into a liquid medium (pH 7.6) composed of KH_2_PO_4_ (1.25 g/L), NaCl (0.1 g/L), (NH_4_)_2_SO_4_ (5 g/L), MgSO_4_ × 7H_2_O (0.625 g/L), and CaCl_2_ × 2H_2_O (0.1 g/L), supplemented with casamino acid (5 g/L) and glucose (20 g/L) as nitrogen and carbon sources, respectively. This medium represented iron (−Fe)- and zinc (−Zn)-depleted conditions. The iron (+Fe)- and zinc (+Zn)-repleted medium contained 50 μM of FeCl_3_ × 6H_2_O and ZnSO_4_ × 7H_2_O. The strains were cultivated as submerged cultures in biological duplicates (37 °C, 190 rpm, 4 days) with a combination of iron and zinc availabilities (−Fe/–Zn, −Fe/+Zn, +Fe/–Zn, and +Fe/+Zn). The supernatant samples were collected at 0, 8, 19, 26, 32, 48, 72, and 96 h and filtered through 0.45 μm LUT Syringe filters (HPST, Prague, Czechia). The biomasses were harvested at the 96th hour, dried, and accurately weighed.

### Extraction of Siderophores

Fifty microliters of corresponding supernatants were spiked with 5 μL of an internal standard ferri-ferrioxamine E (FoxE, 10 μg/mL, Biophore Research Products, Rottenburg, Germany) and extracted once with precooled isopropanol (400 μL). The supernatants were shaken for 0.5 min and stored in a deep freezer for 1 h. Then, samples were centrifuged (4 °C, 10 min, 14,000*g*), and the supernatants were transferred to new 0.5 mL vials and evaporated under reduced pressure (35 °C, 1 h). The extracts were resuspended in 5% acetonitrile (ACN, 100 μL) and submitted to liquid chromatography-mass spectrometry (LC-MS), LC-tandem mass spectrometry (LC-MSMS), and matrix-assisted laser desorption/ionization (MALDI) analysis.

### LC-MS and LC-MSMS Analysis

Resuspended samples were analyzed using a Dionex UltiMate 3000 UHPLC Liquid Chromatograph system (Thermo Fisher Scientific, MA, USA) connected to a 12T solariX Fourier-Transform Ion Cyclotron Resonance mass spectrometer (FTICR-MS, Bruker Daltonics, Boston, MA, USA). Samples were injected (1 μL) onto an ACQUITY HSS T3 C18 analytical column (1.8 μm, 1.0 × 150 mm; Waters, MA, USA) preheated at 40 °C. Analytes were separated within a 13 min gradient elution of 0.1% formic acid in 1% (A) and 99% ACN (B, flow rate: 50 μL/min). The gradient elution was performed as follows: 2% B (0–1 min), 99% B (1–9 min), 99% B (9–10 min), 2% B (10–10.1 min), and 2% B (10.1–13 min). Mass spectra were collected in the electrospray ionization positive-ion mode (ESI+) within a 100–1500 Da mass range. The LC-MSMS-ESI+ mass spectra were obtained by collision-induced dissociation (CID) upon isolating selected cations within a mass range of 7 Da mass range. Fragmentation spectra were collected at a 16 to 18 V collision energy.

All analytes were identified using a combination of accurate mass and MSMS pattern, and the match with retention time was adopted for the ferri-coprogen (Cop) and ferri-Fc standards. Data were quantitatively processed against a six-point external calibration curve within 0.1 and 1000 ng/mL for Cop (used for all coprogens) and Fc using DataAnalysis 6.0 (Bruker Daltonics, MA, USA) and CycloBranch 2.1.35.^34,35^ The LC-MS-ESI+ method validation covered the evaluation of linearity, the limit of detection (LOD) and quantitation (LOQ), trueness, precision, carryover effect, and stability of the retention times. For more details on the LC-MS-ESI+ method, please see Supporting Excel, Figures S1 and S2, and Table S1. All samples were measured in technical duplicates.

### MALDI Analysis

In parallel, 10 μL of the resuspended samples was mixed with the same volume of α-cyano-4-hydroxycinnamic acid (10 mg/mL in 50% ACN/0.1% trifluoroacetic acid) and spotted (1 μL) onto an MTP 384 ground steel MALDI plate. The FTICR-MS was externally calibrated with red phosphorus (1 mg/mL in 50% ACN), providing mass accuracy better than 3 ppm. The positive-ionization mode (MALDI+) and mass range remained the same. Desorption/ionization of the analytes was performed using a SmartBeam II laser (35% laser power, 150 laser shots, and 0.5 kHz frequency). The final mass spectra represented the average of 100 individual scans, randomly collected within a 1.5 mm diameter with a Smart Walk function to acquire reliable data. Data were quantitatively processed against a six-point external calibration curve within 25 and 5000 ng/mL for Cop standard (used for all coprogens) using DataAnalysis 6.0 (Bruker Daltonics, MA, USA) and CycloBranch 2.1.35.^34,35^ The MALDI-MS method validation covered the evaluation of linearity, LOD and LOQ, trueness, and precision. For more details on the MALDI+ method, please see Supporting Information and Table S2. All samples were measured in technical duplicates.

### Ionization Efficiency of Aluminum Coprogen

Fifty microliters of the desferri-coprogen standard (1 μg/mL, Biophore Research Products, Rottenburg, Germany) were spiked with 5 μL of Al_2_(SO_4_)_3_ (1 mg/mL in water) and incubated for 1 h at room temperature. The extraction, LC-MS-ESI+, and MALDI+ analyses remained identical.

### Cultivation of *S. boydii* and Isolation of Novel Scedocyclins

*Sb* (strain CBS 119458) was obtained from the Westerdijk Fungal Biodiversity Institute (Utrecht, The Netherlands). The inoculum preparation and submerged cultivation with optimal growth conditions have been previously reported.^32^ Briefly, *Sb* was cultivated in a liquid Czapek-Dox broth medium (pH 7.3) composed of K_2_HPO_4_ (1 g/L), KCl (0.5 g/L), MgSO_4_ × 7H_2_O (0.5 g/L), NaNO_3_ (2 g/L), FeSO_4_ (0.01 g/L), and saccharose (10 g/L) as a carbon source. The isolation of novel scedocyclins was analogous to that described for pseudacyclins.^32^ Product ion mass spectra were collected using an APEX-Q 9.4T FTICR-MS instrument (Bruker Daltonics, MA, USA) equipped with an Apollo II ESI/MALDI ion source in the positive-ion mode. The product ion mass spectra were generated via CID from the single protonated precursors isolated with a 5 Da mass range in a quadrupolar mass filter preceding the ICR cell.

### Data Treatment and Statistical Evaluation

Data obtained by LC-MS-ESI+, LC-MSMS-ESI+, and MALDI+ were further processed and statistically evaluated using Microsoft Excel 2023 (Microsoft, WA, USA), OriginPro 2021b (OriginLab, MA, USA), and NCSS 23.0.2 software (NCSS, UT, USA). The LC-MS-ESI+ and MALDI+ data are expressed as the arithmetic mean (AVG) ± the standard error of the mean (SEM). The Gaussian distribution of the data set was evaluated by the Shapiro–Wilk test, and the normality assumption was not rejected for the vast majority of collected data. The parametric two-sample t-test was used to evaluate the differences in the siderophore secretion rate under various metal availabilities. The *p*-values ≤ 0.05 were considered statistically significant. Before the correlation analysis, the LC-MS-ESI+ and MALDI+ data were logarithmically transformed using a decadic logarithm. The strength of the linear relationship between these ionization techniques was evaluated using Pearson’s correlation coefficient *r*.

### Mapping of Genes and Proteins Involved in the SIA of *L. prolificans*

The antiSMASH 7.0 tool^36^ was used to screen the biosynthetic gene clusters for secondary metabolites. The identified clusters in the genome of *Lp* strain JHH-5317^28^ were compared with similar clusters of *Sa* (strain IHEM 14462)^25^ and *Af* (strain Af293)^37^ using the KnownClusterBlast feature. The protein similarity was analyzed using the BLASTP or BLASTX algorithm.^38^

## Results and Discussion

### *S. apiospermum* and *L. prolificans* Secrete a Uniform Portfolio of Coprogens

**Figure 1:**
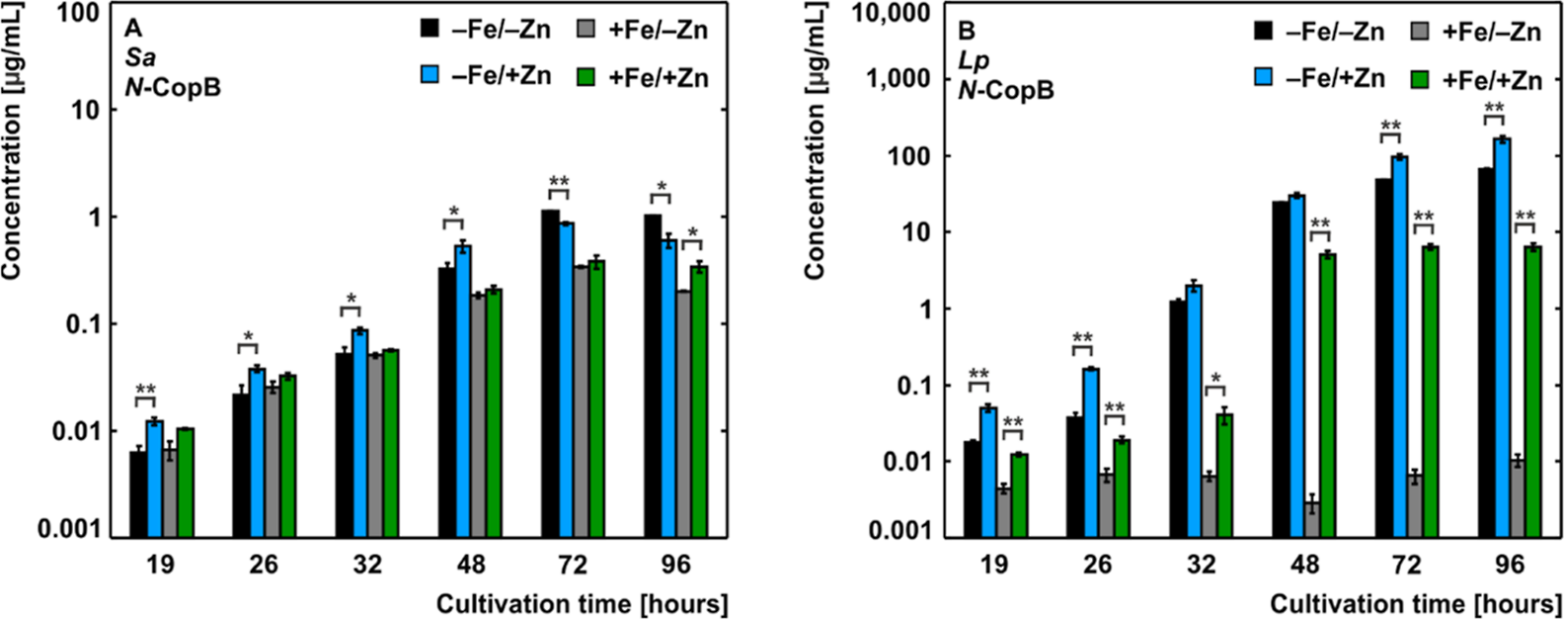
Iron- and zinc-regulated secretion kinetics of *N*-CopB in *Sa* (A) and *Lp* (B). The data are presented as AVG ± SEM (in a decadic logarithm scale) and have been sorted to highlight the regulatory effect of zinc under iron restriction (black and blue) and iron surplus (gray and green). Statistical evaluation: Two-sample t-test on the *p* level ≤0.05 (*) and 0.01 (**).

**Figure 2:**
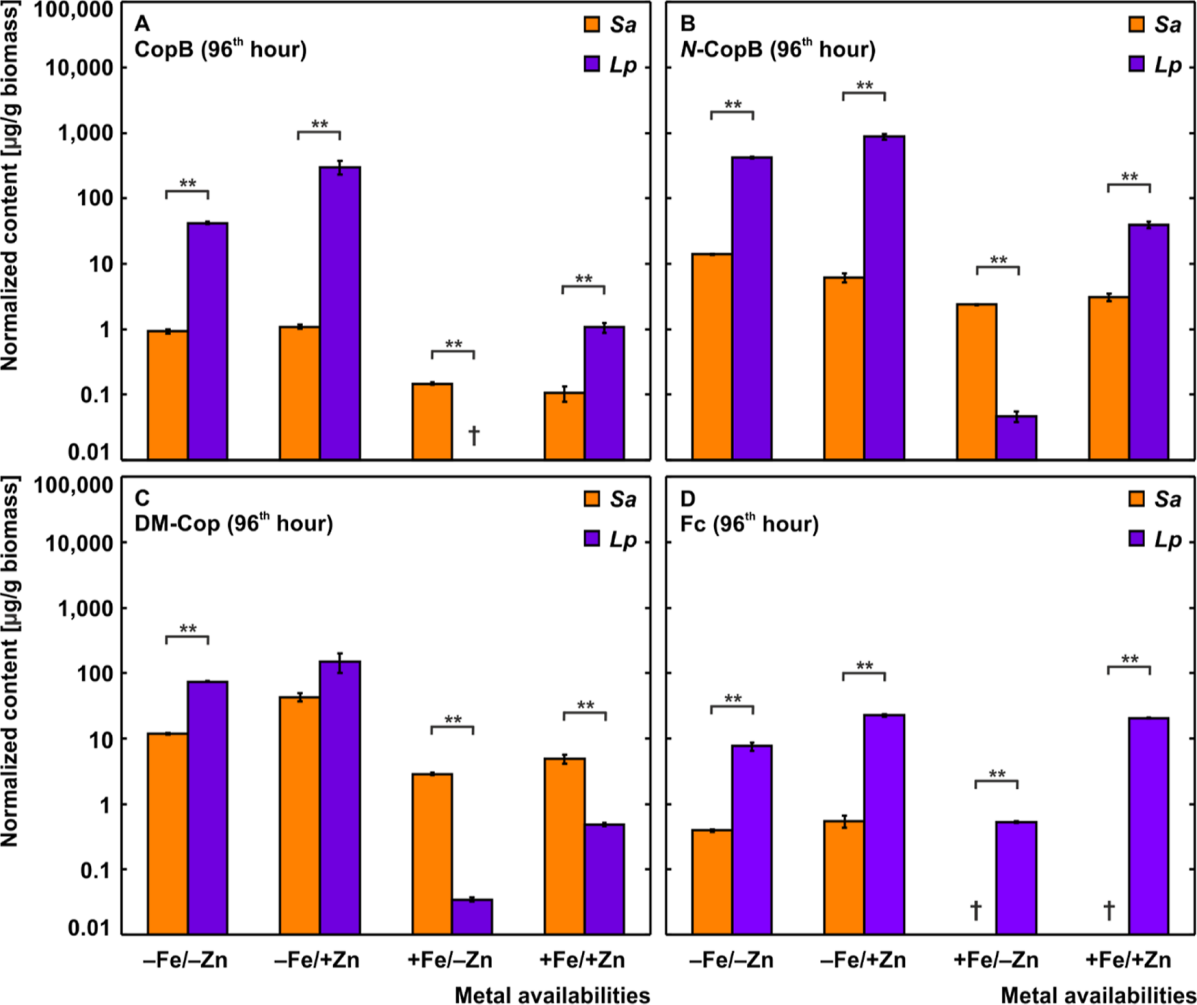
Comparison of the iron- and zinc-regulated specific siderophore production at the 96th hour of cultivation, including CopB (A), *N*-CopB (B), DM-Cop (C), and Fc (D), in *Sa* (orange) and *Lp* (purple). The data were normalized to 1 g of fungal biomass and are presented as AVG ± SEM (in a decadic logarithm scale). Statistical evaluation: Two-sample t-test on the *p* level ≤ 0.01 (**). †: not detected.

**Figure 3:**
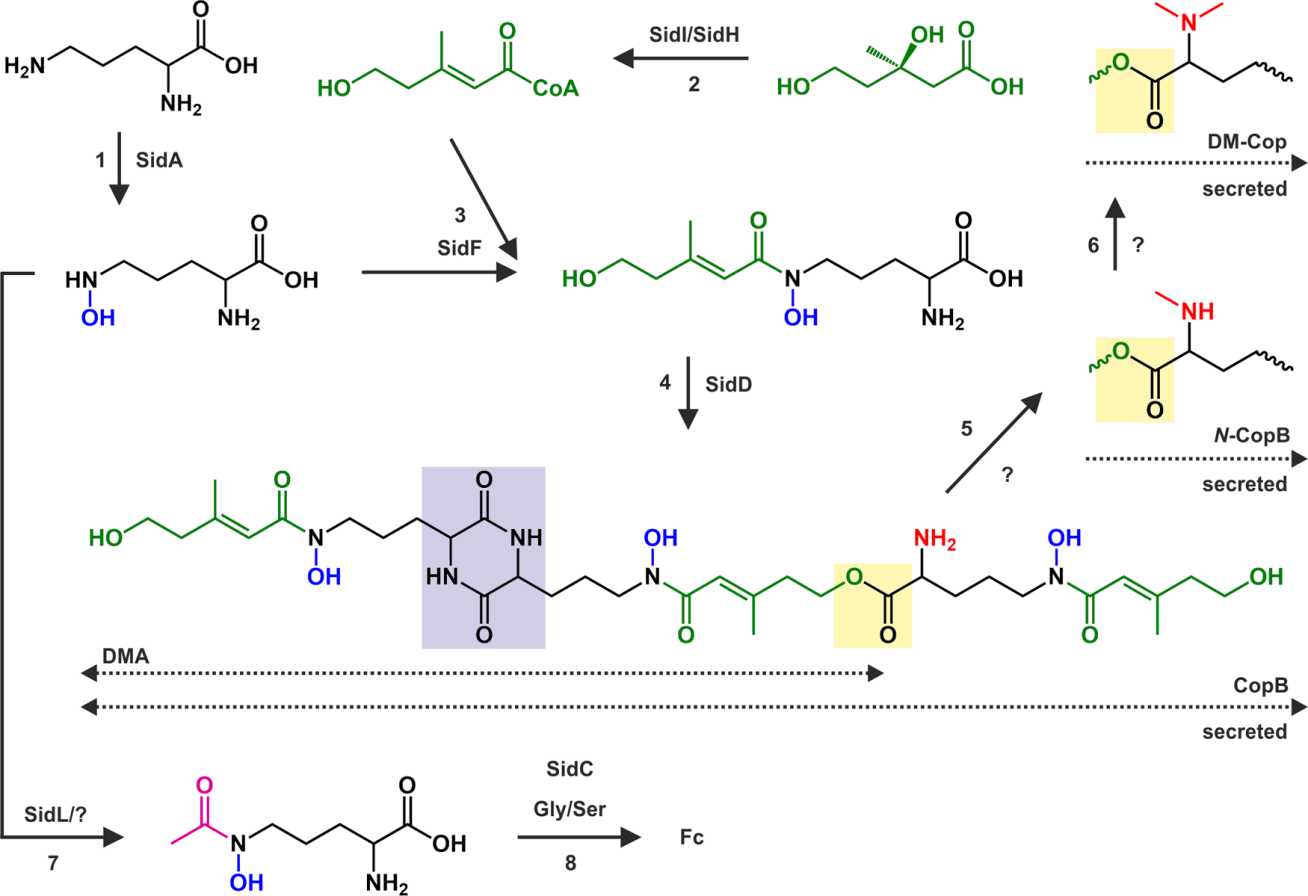
Proposed biosynthesis of CopB, *N*-CopB, DM-Cop, and Fc in *Sa* and *Lp*. During the biosynthesis of the extracellular coprogens (steps 1, 2, 3, 4, 5, and 6), CopB is assembled from three identical subunits, each composed of one hydroxylated (blue) l-ornithine (black) and one anhydromevalonate (green). Two subunits are connected via a diketopiperazine ring (purple background), while the third is assembled through an ester bond (yellow background). A free amine (red) is subsequently methylated and *bis*-methylated with unknown (?) enzymes to create *N*-CopB and DM-Cop, respectively. The biosynthesis of Fc (steps 1, 7, and 8) requires acetylation (pink) of three hydroxylated l-ornithines followed by their connection with two Gly and one Ser.

*Sa* and *Lp* were cultivated under distinct metal variance conditions, and their supernatants were screened for the secretion kinetics of extracellular siderophores. Regardless of the iron and zinc content, both strains produced the same spectrum of coprogens: coprogen B (CopB), *N*-CopB, and dimethyl-coprogen (DM-Cop), with *N*-CopB being the earliest and most abundant secreted molecule appearing 19th hour post-inoculation (Figure 1A,B). This speed of siderophore mobilization is comparable to the TafC appearance in *Af* under comparable cultivation conditions.^20^ Both strains displayed continuously increasing secretion of coprogens, reaching their maximum between the 72nd and 96th hour of cultivation (Figures S3 and S4). Based on the coprogen amount produced by 1 g of fungal biomass, *Lp* demonstrated approximately 45 times more powerful secretion machinery for CopB than *Sa* under iron and zinc limitation (Figure 2A). In the postulated biosynthesis of fungal coprogens (Figure 3), CopB is the first assembled structure, which further undergoes single and double *N*^2^-methylation or single *N*^2^-acetylation to produce the corresponding methylated analogues or Cop, respectively.^39^ In *Sa*, this methylation was evidenced by the lowest concentration of CopB with a preferential secretion of *N*-CopB, culminating at the 72nd hour post-inoculation, followed by the subsequent shift to DM-Cop during iron restriction (Figure S3). In contrast, under the same conditions, *Lp* preferred the production of *N*-CopB over DM-Cop, with its highest secretion at the 96th hour (Figures 2B,C and S4). Compared to *Af*,^37^ the genomes of *Sa* and *Lp* contain all the putative enzymes required for the biosynthesis of the extracellular siderophores (Table S3), except for the transacetylase SidG, which could explain the absence of Cop.

### Iron Surplus Reduces the Species-Specific Secretion of Coprogens

In both strains, an iron surplus reduced the specific production of coprogens but did not affect the preferential occurrence of their methylated analogues (Figure 2A–C). Assuming the species-specific production of preferred *N*-CopB, excess iron reduced its secretion by 83% and 99% in *Sa* and *Lp*, respectively (Figure 2B). This significant downregulation of siderophore biosynthesis indicates the presence of iron-sensing transcriptional factors that protect fungal strains from iron overdose.^19^ Indeed, the genome of *Sa* and *Lp* contains putative homologues of the transcription factors SreA and HapX (Table S3).

### Zinc Promotes the Biosynthesis of Coprogens

Assuming the species-specific secretion of coprogens, a statistically significant (*p* ≤ 0.01) upregulation by zinc was recorded for CopB, *N*-CopB, and DM-Cop in *Lp*, irrespective of iron availability (Figure S5). In *Sa*, the same effect of enhanced secretion was observed for DM-Cop, followed by *N*-CopB under iron restriction for up to the 48th hour of incubation (Figure S5). On the transcriptional level, zinc-inducible SreA represses siderophore production through transcriptional activator HapX under iron sufficiency;^19^ therefore, secretion of coprogens with the combination of iron and zinc sufficiency indicates the involvement of other zinc-inducible transcription factors such as AcuM and AtrR.^30,31^ These transcription factors are putatively present in the genomes of *Sa* and *Lp* as well (Table S3). Compared to iron sufficiency only, a statistically significant (*p* ≤ 0.01) specific production of the coprogens was observed in *Lp* under iron and zinc surplus (Figure 2A–C). This phenomenon is likely due to suppressing the SreA activity by activating the zinc-inducible AcuM, which induces HapX to stimulate gene expression in siderophore-mediated iron uptake.^30^ Simultaneously, the enhanced coprogen secretion is activated by the zinc-inducible transcription factor AtrR.^31^

### Extracellular Detection of Fc

The faster mobilization of coprogens by growth-promoting biomass (Supporting Excel) was accompanied by the delayed detection of extracellular Fc in both strains (Figure S6), confirming its pan-fungal role as the primarily intracellular iron keeper.^29^ In *Lp*, the secretion kinetics of Fc was upregulated (*p* ≤ 0.01) by zinc regardless of iron presence, copying the secretion kinetics of extracellular coprogens (Figure S5). In *Sa*, Fc was detected under iron restriction only. Notably, *Lp* showed more significant specific production of Fc under all metal stress combinations than *Sa* (Figure 2D).

### *S. boydii* Produces Novel Cyclic Peptides

**Figure 4:**
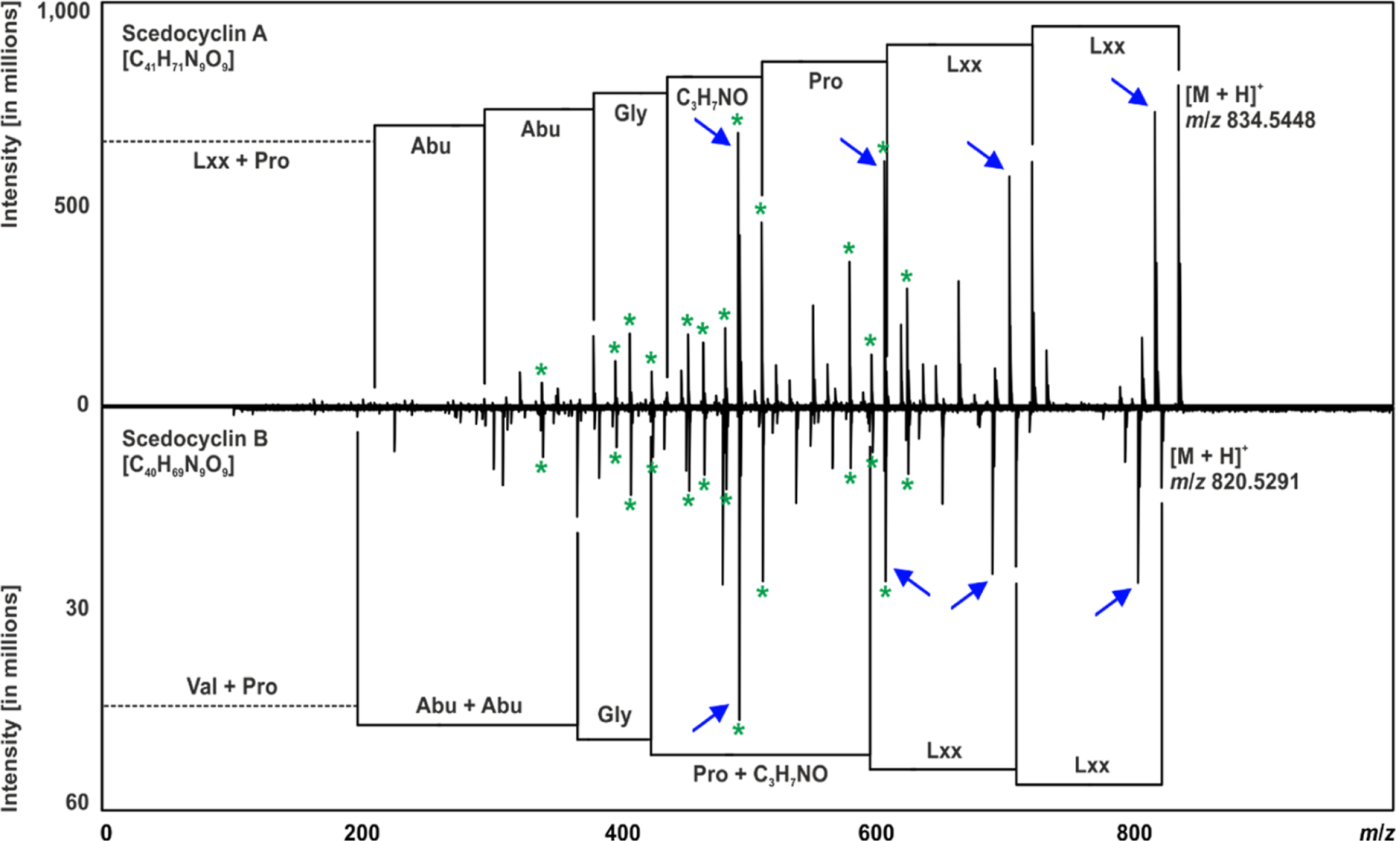
Comparison of the CID tandem mass spectra collected for Scedocyclin A and Scedocyclin B. The spectra were annotated assuming the preferential fragmentation due to the Pro hypercleavage, resulting in the presence of identical *b* ions (green asterisks) and dehydrated *b* ions (blue arrows).

**Figure 5:**
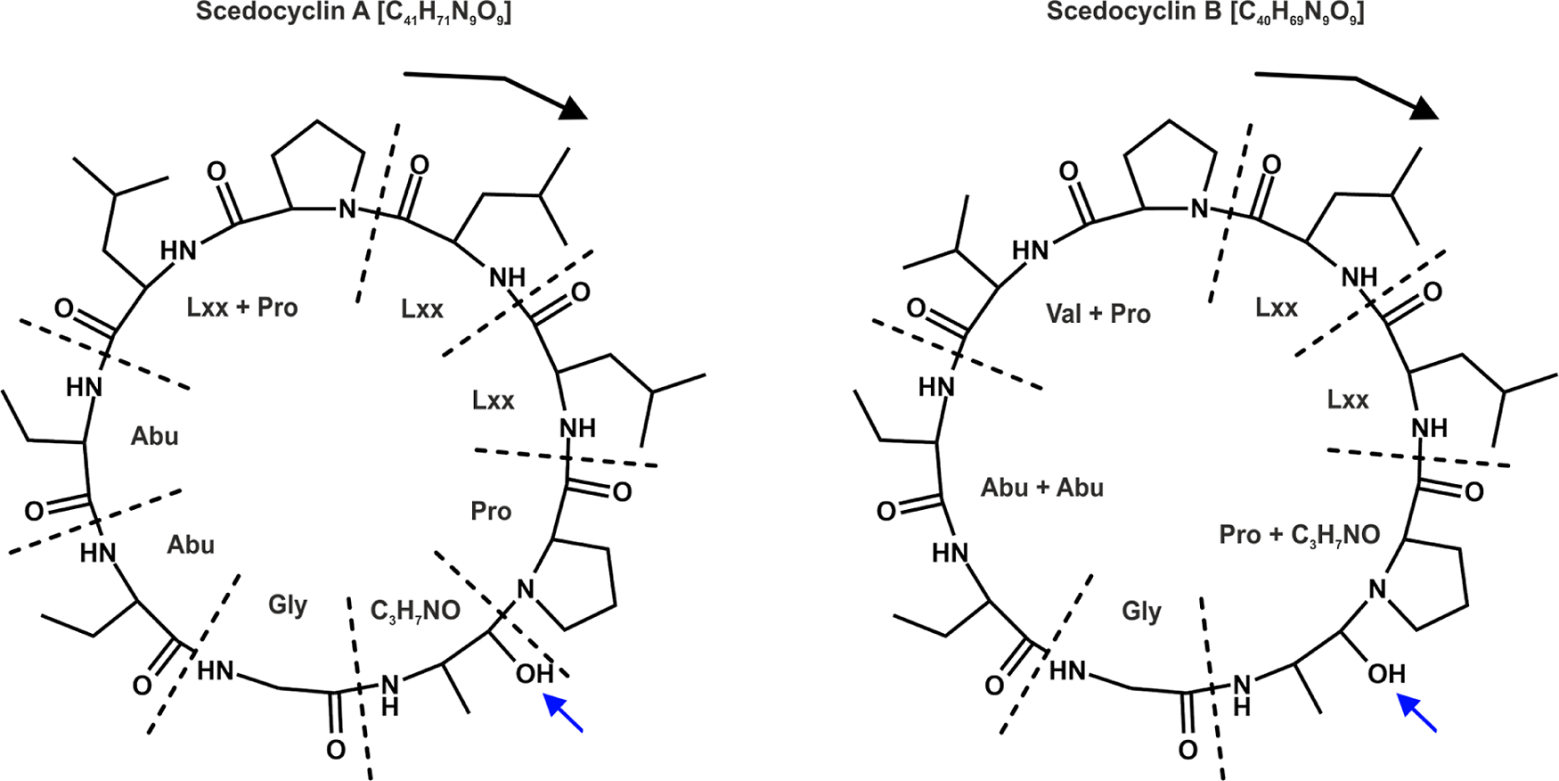
Proposed molecular structures of Scedocyclin A and Scedocyclin B upon the *de novo* analysis of the CID mass spectra using CycloBranch. The black arrow denotes the primary fragmentation mechanism due to the Pro hypercleavage. The blue arrow points to the hydroxyl group, which was most likely integrated within C_3_H_7_NO due to the presence of dehydrated *b* ions.

CycloBranch^34,35^ was used to characterize two novel cyclic peptides, which we called scedocyclins, with *m*/*z* 834.5448 (Scedocyclin A) and *m*/*z* 820.5291 (Scedocyclin B), revealing their elemental compositions [C_41_H_71_N_9_O_9_ + H]^+^ and [C_40_H_69_N_9_O_9_ + H]^+^, respectively (Figure S7). The following *de novo* analysis of CID mass spectra discovered multiple overlapping series of *b* ions (Table S4), indicating a cyclic peptide structure. The probable structures could be composed of nine building blocks, including leucine, isoleucine, or a similar isobaric amino acid (Lxx), valine (Val), proline (Pro), glycine (Gly), α-aminobutyric acid (Abu), and an unknown block with the residual molecular formula of C_3_H_7_NO. Assuming the primary fragmentation mechanism was due to the Pro hypercleavage,^40^ the CID spectra were annotated (Figure 4) and revealed the tentative sequence for Scedocyclin A as cyclo(Pro-Lxx-Abu-Abu-Gly-C_3_H_7_NO-Pro-Lxx-Lxx) and for Scedocyclin B as cyclo(Pro-Val-Abu-Abu-Gly-C_3_H_7_NO-Pro-Lxx-Lxx) (Figure 5), revealing their difference in the methylene group most likely due to the substitution of Lxx in Scedocyclin A with Val in Scedocyclin B, respectively. The presence of C_3_H_7_NO was predicted due to the detection of dehydrated *b* ions, as other blocks did not tend to lose water molecules. Note that the pairs of residues Abu + Abu, Gly + Lxx, and Pro + C_3_H_7_NO formed isomers with equal molecular formula C_8_H_14_N_2_O_2_. Of note, these peptides were found on the intact *Sb* spores but were never characterized further.^33^ They may possess an obscure role in conidial germination and require further study.

### Comparison of Ionization Techniques for the MS Detection of Coprogens

**Figure 6:**
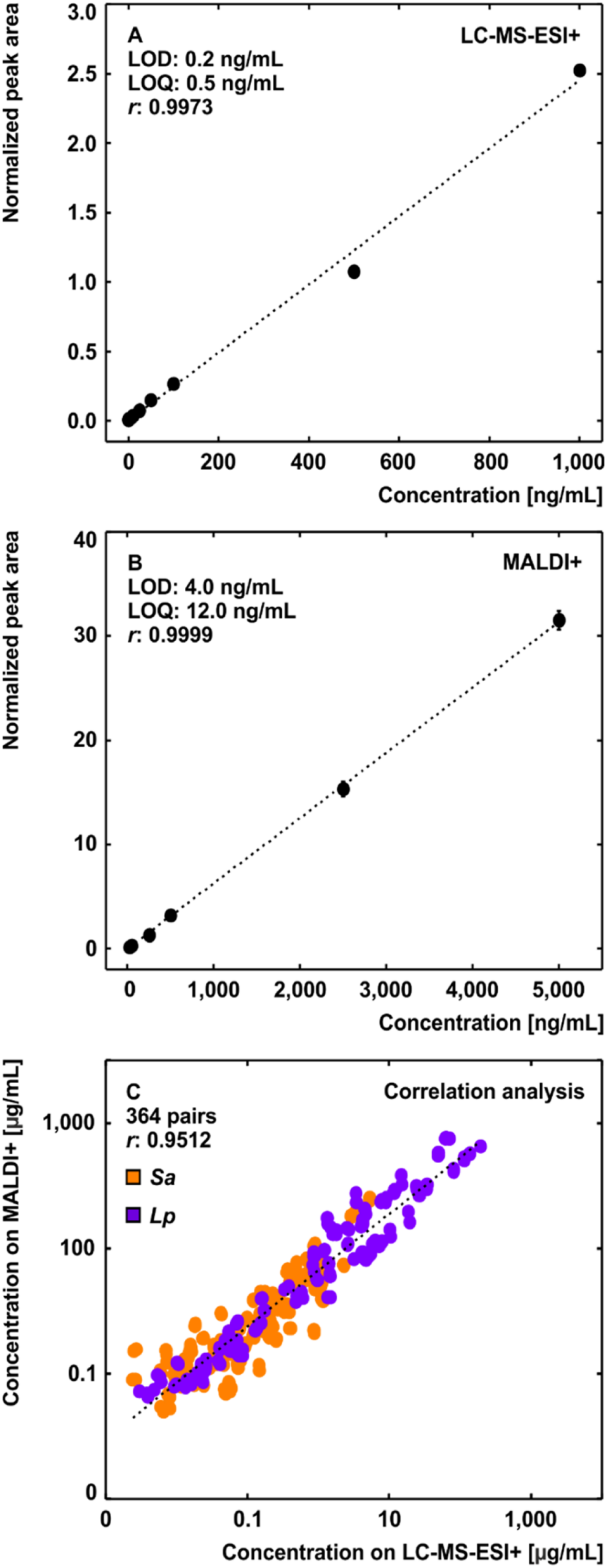
Comparison of the instrumental detection limits of the Cop standard using LC-MS-ESI+ (A) and MALDI+ (B), and the correlation analysis between these ionization techniques (C), favoring detection through MALDI+ due to the elevated ionization efficiency of coprogens in their corresponding aluminum(III) complexes.

Mass spectrometry-based diagnostics utilizing fungal siderophores provide a sensitive method for detecting invasive mycoses.^41^ Siderophores are predominantly detected in their corresponding ferri forms in patients.^20^ The LC-MS-ESI+ analysis of the Cop standard recovered from the cultivation medium matrix revealed a LOD and LOQ of 0.2 and 0.5 ng/mL, respectively (Figure 6A). In contrast, direct use of MALDI+ (Figure 6B) provided 20 and 24× higher LOD (4.0 ng/mL or 4.9 fmol/spot) and LOQ (12.0 ng/mL or 14.6 fmol/spot), respectively. The correlation analysis (Figure 6C), which compared all quantitative results of coprogens detected during the *in vitro* cultivation study of *Sa* and *Lp*, revealed a strong linear relationship (*r* = 0.9512, *p* ≤ 0.01) between the ionization techniques used. This linearity represents a strong argument for the future applications of quantitative Biotyper (MALDI-time-of-flight instruments) in medical mycology.^42^ Furthermore, the method comparison revealed a quantitative shift in favor of MALDI+ due to the increased ionization efficiency of coprogens in their aluminum complexes, further confirmed by comparing the ionization ratios between the ferri- and aluminum-coprogen (Supporting Excel). Saturation of coprogens with aluminum may further improve the diagnostic sensitivity on Biotyper instruments.

## Conclusions

*Sa* and *Lp* are opportunistic filamentous fungi for humans, and their detection is still limited to direct examination combined with culture. We showed here that *Sa* and *Lp* produced the same siderophores, including CopB, *N*-CopB, DM-Cop, and Fc, with *N*-CopB being the earliest and preferentially secreted coprogen. Compared to *Sa*, *Lp* showed a more powerful producing capacity of all coprogens under iron restrictions, presumably mirroring its higher virulence. Furthermore, the biosynthesis of siderophores was supported by the zinc availability. Due to this enormous biosynthetic machinery, coprogens represent fungal virulence factors, reflecting active pathogen proliferation and potential clinical biomarkers of invasive scedosporiosis and lomentosporiosis, which could also be routinely detected in next-generation Biotyper applications. In contrast to the coprogens, two novel scedocyclins were associated with *Sb* conidia and characterized using our in-house CycloBranch software, revealing their structural difference most likely due to the substitution of Lxx with Val.

## Supporting Information Available

Representative LC-MS chromatograms and MSMS spectra of the detected coprogens, secretion kinetics of coprogens and ferricrocin in *S. apiospermum* and *L. prolificans*, CycloBranch setup window, and analytical overview and method validation in ESI and MALDI, as well as protein and fragment ion lists relevant to the main manuscript (PDF)
LC-MS-MALDI method validation, LC-MS-ESI+ data, MALDI+ data, correlation analysis, and LC-MS two-sample t-tests (XLSX)

## Abbreviations Used

Abu: α-aminobutyric acid
ACN: acetonitrile
*Af*: *Aspergillus fumigatus*
AVG: arithmetic mean
CF: cystic fibrosis
CID: collision-induced dissociation
Cop: ferri-coprogen
CopB: coprogen B
DMA: dimerumic acid
DM-Cop: dimethyl-coprogen
Fc: ferri-ferricrocin
FoxE: ferri-ferrioxamine E
FTICR-MS: Fourier-Transform Ion Cyclotron Resonance mass spectrometer
Gly: glycine
LC-MS-ESI+: liquid chromatography-mass spectrometry analysis detection using electrospray ionization in the positive-ion mode
LOD: instrumental limit of detection
LOQ: instrumental limit of quantitation
*Lp*: *Lomentospora prolificans*
Lxx: leucine, isoleucine, or a similar isobaric amino acid
MALDI+: matrix-assisted laser desorption/ionization in the positive-ion mode
*N*-CopB: *N*^α^-methyl-coprogen B
Pro: proline
*r*: Pearson’s correlation coefficient
*Sa*: *Scedosporium apiospermum*
*Sb*: *Scedosporium boydii*
SEM: standard error of the mean
Ser: serine
SIA: siderophore-mediated iron acquisition
TafC: triacetylfusarinine C
Val: valine
