# Supplementary material for: Exploring the Siderophore Portfolio for Mass Spectrometry-Based Diagnosis of Scedosporiosis and Lomentosporiosis
Source: ACS Omega. 2024 Oct 23;9(44):44815–24. doi: 10.1021/acsomega.4c08257 (PMC11541790; doi:10.1021/acsomega.4c08257)
Supplement: Supplementary file 1 — ao4c08257_si_001.pdf [file ao4c08257_si_001.pdf]

SUPPORTING INFORMATION

Exploring the Siderophore Portfolio for Mass Spectrometry-Based Diagnosis of Scedosporiosis and Lomentosporiosis

Jiří Houšť[1,2], Andrea Palyzová[1], Tomáš Pluháček[1,2], Jiří Novák[1,3], Helena Marešová[1], Petr Hubáček[4], Radim Dobiáš[5], David A. Stevens[6], Hélène Guegan[7], Jean-Pierre Gangneux[7], Vladimír Havlíček[1,2*]

[1]Laboratory of Molecular Structure Characterization, Institute of Microbiology of the Czech Academy of Sciences, Vídeňská 1083, 142 00 Prague, Czechia; E-mail: jiri.houst@biomed.cas.cz, palyzova@biomed.cas.cz, tomas.pluhacek@biomed.cas.cz, jiri.novak@biomed.cas.cz, maresova@biomed.cas.cz, Vlhavlic@biomed.cas.cz

[2]Department of Analytical Chemistry, Faculty of Science, Palacký University in Olomouc, 17. listopadu 1192/12, 779 00 Olomouc, Czechia

[3]Department of Software Engineering, Faculty of Information Technology, Czech Technical University in Prague, Thákurova 9, 160 00 Prague, Czechia

[4]Department of Medical Microbiology, 2nd Faculty of Medicine, Charles University and Motol University Hospital, V Úvalu 84, 150 06 Prague, Czechia; E-mail: petr.hubacek@fnmotol.cz

[5]Department of Bacteriology and Mycology, National Reference Laboratory for Mycological Diagnostics, Public Health Institute, Partyzánské náměstí 2633/7, 702 22 Ostrava, Czechia; Institute of Laboratory Medicine, Faculty of Medicine, University of Ostrava, Syllabova 19, 703 00 Ostrava, Czechia; E-mail: radim.dobias@zuova.cz

[6]Division of Infectious Diseases and Geographic Medicine, Stanford University School of Medicine, Stanford, 291 Campus Drive, CA 95128, USA; E-mail: stevens@stanford.edu

[7]Division of Parasitology and Mycology, Rennes University Hospital, 2 Rue Henri le Guilloux, 35033 Rennes, France; E-mail: helene.guegan@univ-rennes.fr, jean-pierre.gangneux@univ-rennes.fr

Table of Content

Figure/Table	Name	Page
Figure S1	Representative LC-MS chromatograms of the detected analytes.	S2
Figure S2	Representative MSMS spectra of the detected coprogens.	S3
Figure S3	Secretion kinetics of coprogens in *S. apiospermum*.	S4
Figure S4	Secretion kinetics of coprogens in *L. prolificans*.	S5
Figure S5	Secretion kinetics of coprogens in *Sa* and *Lp*.	S6
Figure S6	Secretion kinetics of Fc in *Sa* and *Lp*.	S7
Figure S7	CycloBranch set up windows for generating elemental formula and isotope profiling.	S8
Table S1	Overview of the detected analytes by LC-MS-ESI+ and the LC-MS-ESI+ method validation for the detection of Cop.	S9
Table S2	Overview of the detected analytes by MALDI+ and the MALDI+ method validation for the detection of Cop.	S10
Table S3	Overview of the proteins involved in the SIA of *Lp*, *Sa*, and *Af*.	S11
Table S4	Overview of the *b*-ion series of Scedocyclin A and Scedocyclin B.	S12

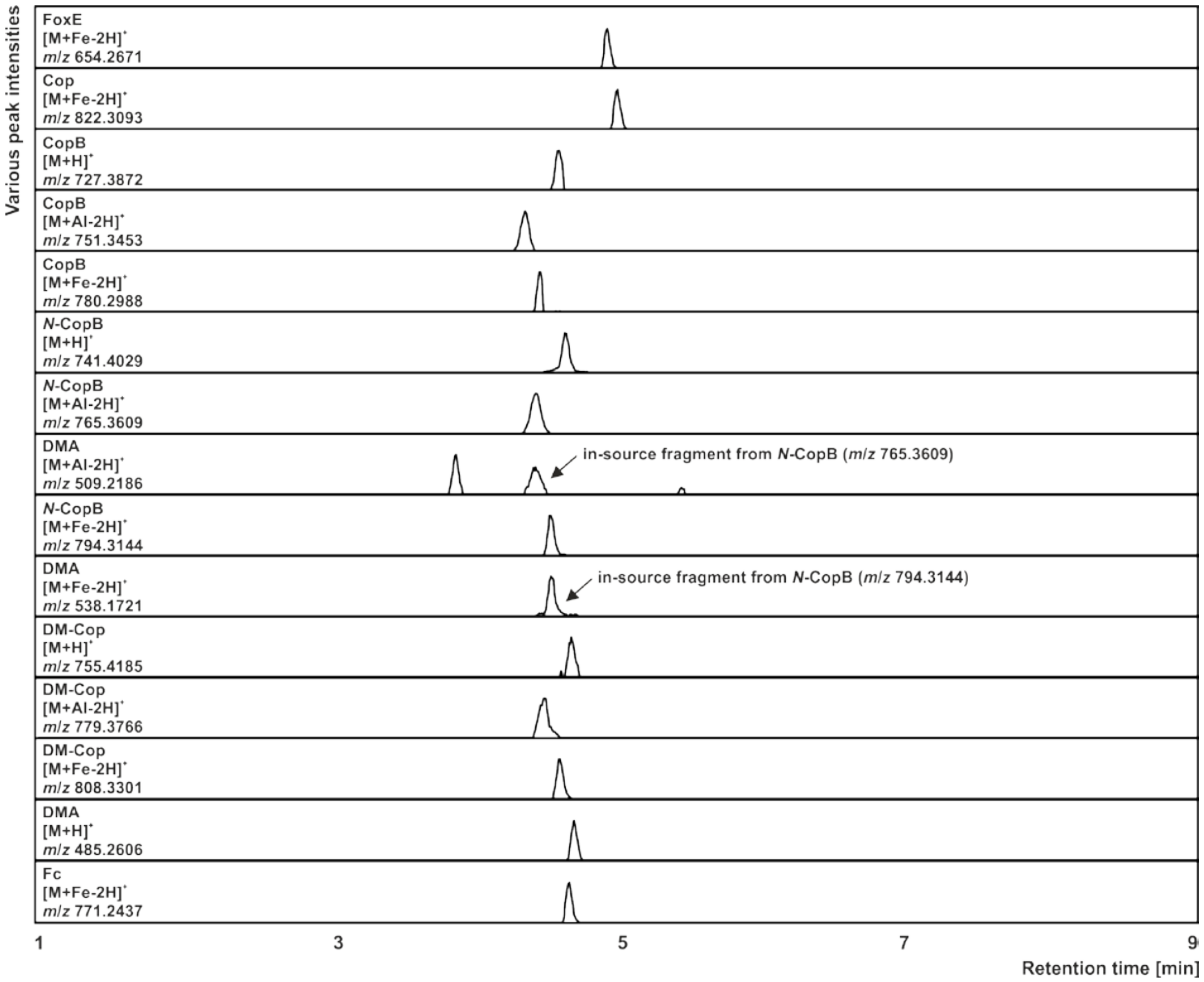

Figure S1. Representative LC-MS chromatograms of the detected analytes. Extracted ion chromatograms were generated for the single protonated non-metallic, iron(III), and aluminium(III) complexes of FoxE, Cop, CopB, *N*-CopB, DM-Cop, DMA, and Fc with a 0.005 Da mass spectral width. The ion chromatograms of FoxE and Cop represent their detection in a 1 000 ng/mL calibration standard. The rest represent their detection in the supernatant of *Lp* collected at the 96th hour under –Fe/+Zn condition.

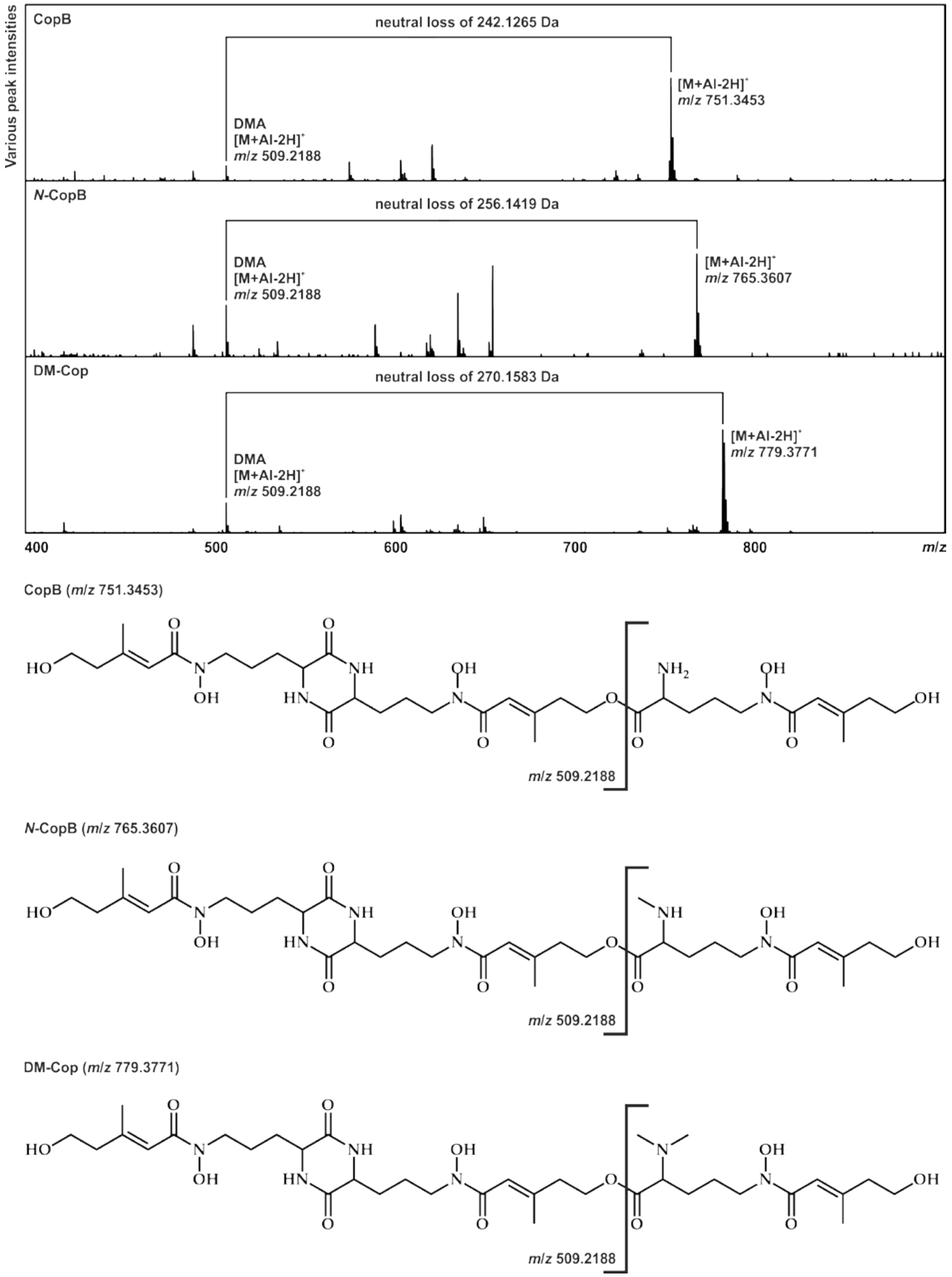

Figure S2. Representative MSMS spectra of the detected coprogens. The aluminium(III) complexes of CopB, *N*-CopB, and DM-Cop fragmented in their ester bonds and provided the neutral losses of 242.1265, 256.1419, and 270.1583 Da, respectively. As a result, DMA was detected in its aluminium(III) complex (*m/z* 509.2188). The mass difference (14.0157 Da) between the observed neutral losses corresponds to the methylene group located as a methyl group at the N^2 position. Molecular structures are drawn without chelated aluminium.

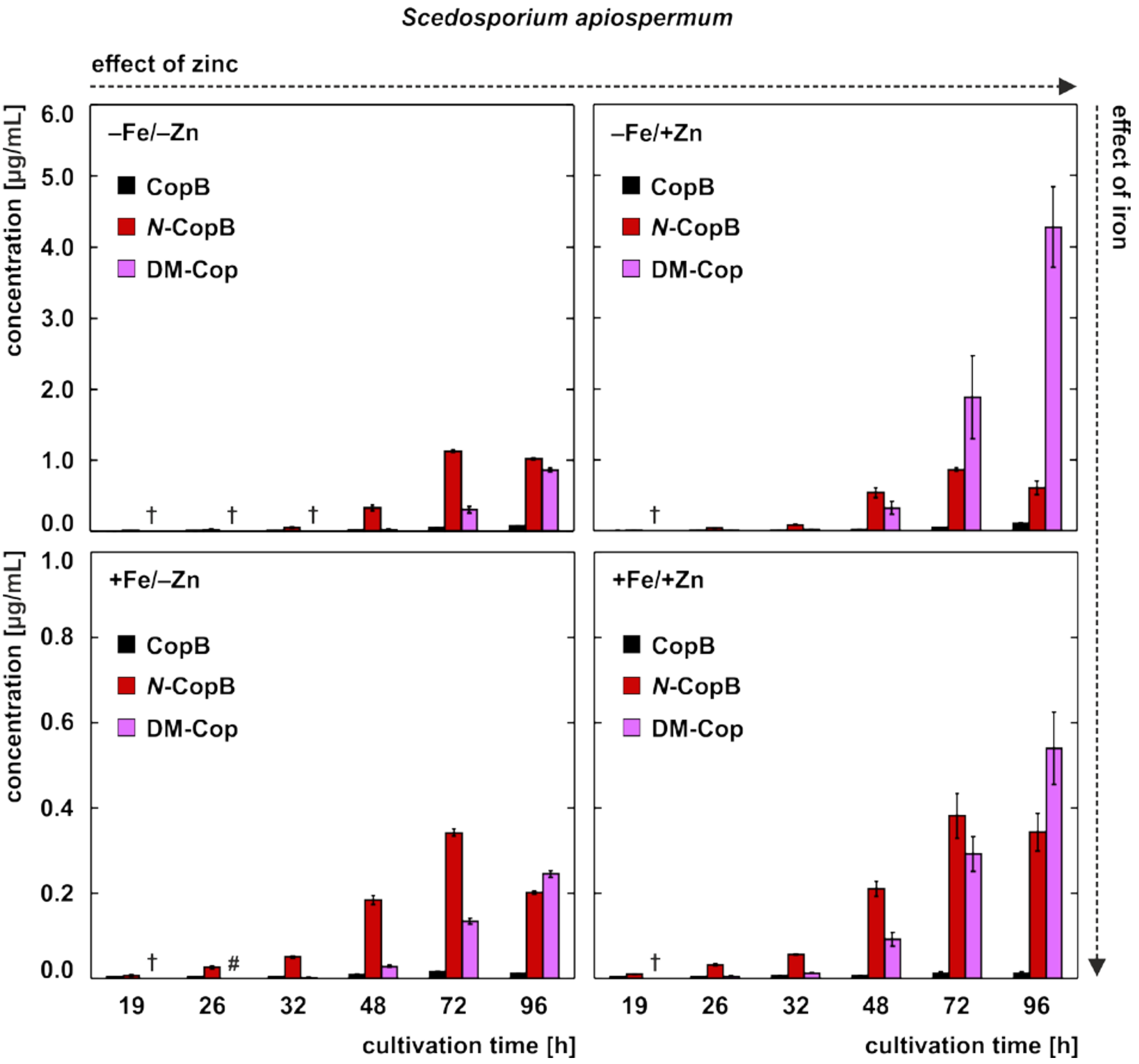

Figure S3. Secretion kinetics of coprogens in *S. apiospermum*. Data are presented as AVG ± SEM and have been sorted to highlight the N^2-methylation kinetics of CopB under various metal availabilities. Data concerning detection of DMA were excluded due to the assumption that DMA was most likely a by-product of the corresponding coprogens upon ester hydrolysis. †: not detected, #: detected.

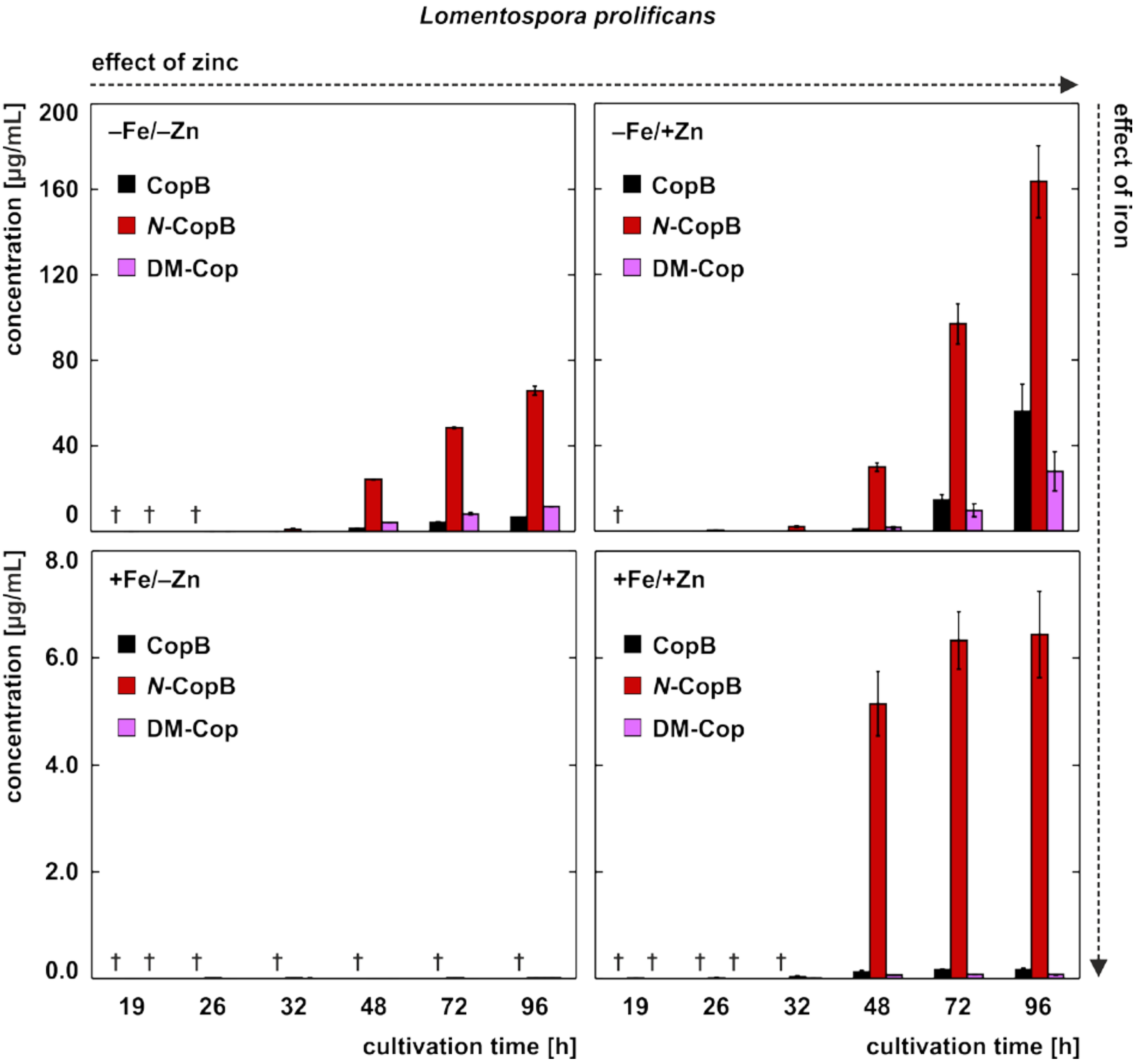

Figure S4. Secretion kinetics of coprogens in *L. prolificans*. Data are presented as AVG ± SEM and have been sorted to highlight the N^2-methylation kinetics of CopB under various metal availabilities. Data concerning detection of DMA were excluded due to the assumption that DMA was most likely a by-product of the corresponding coprogens upon ester hydrolysis. †: not detected.

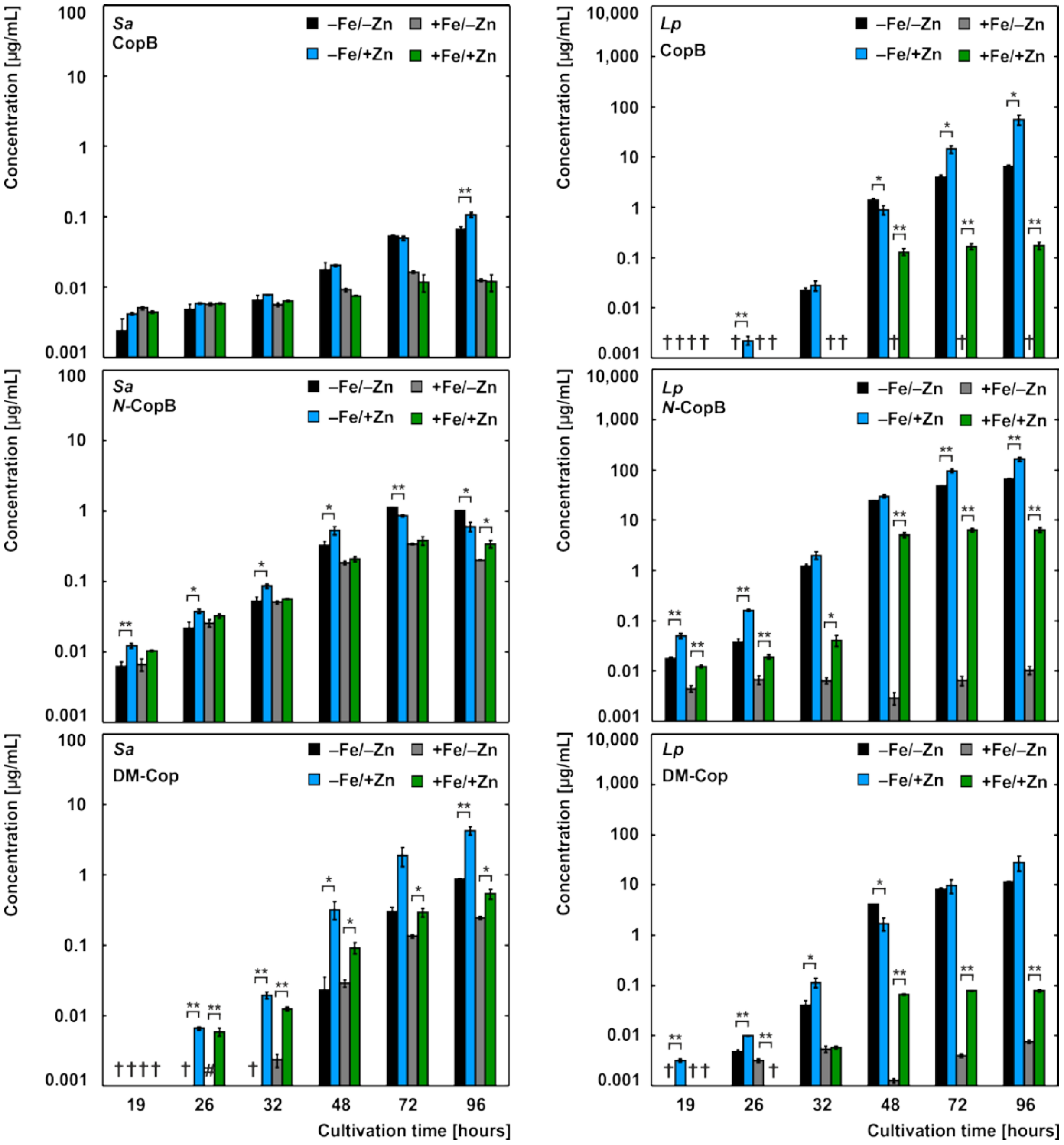

Figure S5. Comparison of the secretion kinetics of coprogens between *Sa* and *Lp*. Data are presented as AVG ± SEM (in a decadic logarithm scale) and have been sorted to highlight the regulatory effect of zinc under iron restriction (black and blue) and iron surplus (grey and green). Data concerning detection of DMA were excluded due to the assumption that DMA was most likely a by-product of the corresponding coprogens upon ester hydrolysis. Statistical evaluation: Two Sample T-Teston the p-level ≤ 0.05 (*) and 0.01 (**). †: not detected, #: detected.

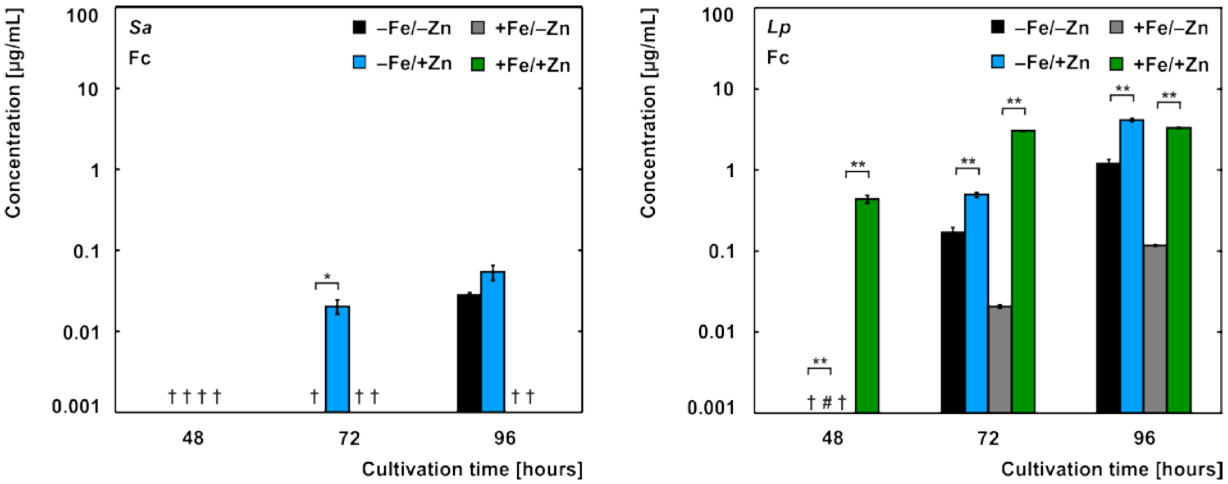

Figure S6. Secretion kinetics of Fc in *Sa* and *Lp*. Data are presented as AVG ± SEM (in a decadic logarithm scale) and have been sorted to highlight the regulatory effect of zinc under iron restriction (black and blue) and iron surplus (grey and green). Statistical evaluation: Two Sample T-Test on the *p*-level ≤ 0.05 (*) and 0.01 (**). †: not detected, #: detected.

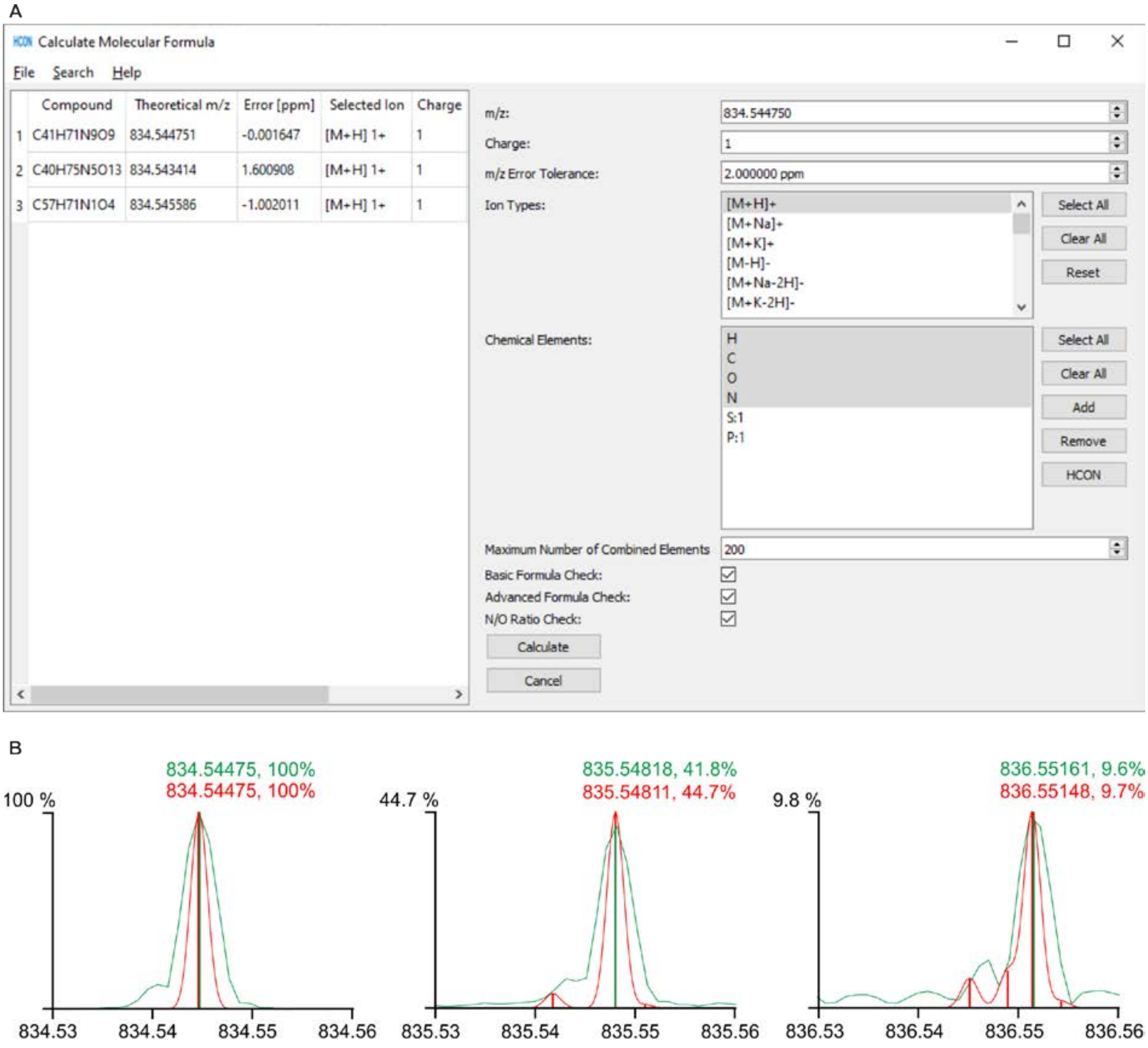

	Theoretical m/z	Theoretical Relative Intensity [%]	Summary Formula
1	834.544751	100.000000	1+ C41 H72 N9 O9 ;
2	835.541786	3.324233	1+ C41 H72 N8 15N O9 ;
3	835.548113	44.687319	1+ C40 13C H72 N9 O9 [99.23%]; 1+ C41 H72 N9 O8 17O [0.77%];
4	835.551028	0.828095	1+ C41 H71 2H N9 O9 ;
5	836.545141	1.474114	1+ C40 13C H72 N8 15N O9 ;
6	836.548997	1.849494	1+ C41 H72 N9 O8 18O ;
7	836.551475	9.744386	1+ C39 13C2 H72 N9 O9 [98.44%]; 1+ C40 13C H72 N9 O8 17O [1.56%];
8	836.554383	0.367215	1+ C40 13C H71 2H N9 O9 ;

Figure S7. CycloBranch set up windows for generating elemental formulas and isotope profiling. In screenshot (**A**), three theoretical elemental formulas were generated within a 2-ppm precision window, considering combinations of hydrogen, carbon, oxygen, and nitrogen with up to 200 atoms in a molecular formula. Scedocyclin A was found to have the composition of $C_{41}H_{71}N_9O_9$ with the lowest difference from the collected accurate mass. Collage (**B**) of theoretical (red) and collected (green) isotopic profile screenshots, which correspond to the protonated molecular cluster of Scedocyclin A.

Table S1. Overview of the detected analytes by LC-MS-ESI+ and the LC-MS-ESI+ method validation for the detection of Cop.

**Overview of the detected analytes by LC-MS-ESI+**					
**Analyte**	**Ion form**	**Retention time [min]**	**Calculated *m/z***	**Measured *m/z***	**Error [ppm]**
FoxE $C_{27}H_{48}N_6O_9$	$[M+Fe-2H]^+$	4.95	654.2671	654.2667	-0.6
Cop $C_{35}H_{56}N_6O_{13}$	$[M+Fe-2H]^+$	5.02	822.3093	822.3086	-0.9
CopB $C_{33}H_{54}N_6O_{12}$	$[M+H]^+$	4.59	727.3872	727.3874	0.3
	$[M+Al-2H]^+$	4.36	751.3453	751.3453	0.0
	$[M+Fe-2H]^+$	4.46	780.2988	780.2966	-2.8
N-CopB $C_{34}H_{56}N_6O_{12}$	$[M+H]^+$	4.64	741.4029	741.4028	-0.1
	$[M+Al-2H]^+$	4.44	765.3609	765.3607	-0.3
	$[M+Fe-2H]^+$	4.54	794.3144	794.3142	-0.3
DM-Cop $C_{35}H_{58}N_6O_{12}$	$[M+H]^+$	4.68	755.4185	755.4185	0.0
	$[M+Al-2H]^+$	4.49	779.3766	779.3771	0.6
	$[M+Fe-2H]^+$	4.61	808.3301	808.3308	0.9
DMA $C_{22}H_{36}N_4O_8$	$[M+H]^+$	4.71	485.2606	485.2607	0.2
	$[M+Al-2H]^+$	3.88	509.2186	509.2188	0.4
Fc $C_{28}H_{47}N_9O_{13}$	$[M+Fe-2H]^+$	4.70	771.2481	771.2464	-2.2

**LC-MS-ESI+ Method Validation for the Detection of Cop**	
**Validation Parameter**	**Results**
Linearity – Pearson's r	0.9973
LOD [ng/mL]	0.2
LOQ [ng/mL]	0.5
Precision 1 (5 ng/mL; expressed as relative standard deviation in %)	1
Precision 2 (100 ng/mL; expressed as relative standard deviation in %)	1
Precision 3 (500 ng/mL; expressed as relative standard deviation in %)	1
Trueness 1 (5 ng/mL; expressed as recovery of Cop in %)	102
Trueness 2 (100 ng/mL; expressed as recovery of Cop in %)	83
Trueness 3 (500 ng/mL; expressed as recovery of Cop in %)	81
Carry-Over Effect	≤ LOD
Stability of the retention time [min] of Cop (October 2023)	4.99
Stability of the retention time [min] of Cop (October 2023)	5.02

Table S2. Overview of the detected analytes by MALDI+ and the MALDI+ method validation for the detection of Cop.

Overview of the detected analytes by MALDI+					
**Analyte**	**Ion form**	**Retention time [min]**	**Calculated *m/z***	**Measured *m/z***	**Error [ppm]**
FoxE $C_{27}H_{48}N_6O_9$	$[M+Fe-2H]^+$	–	654.2671	654.2657	-2.1
Cop $C_{35}H_{56}N_6O_{13}$	$[M+Fe-2H]^+$	–	822.3093	822.3102	1.1
CopB $C_{33}H_{54}N_6O_{12}$	$[M+H]^+$	–	727.3872	727.3888	2.2
	$[M+Al-2H]^+$	–	751.3453	751.3465	1.6
	$[M+Fe-2H]^+$	–	780.2988	780.2998	1.3
N-CopB $C_{34}H_{56}N_6O_{12}$	$[M+H]^+$	–	741.4029	741.4036	0.9
	$[M+Al-2H]^+$	–	765.3609	765.3616	0.9
	$[M+Fe-2H]^+$	–	794.3144	794.3149	0.6
DM-Cop $C_{35}H_{58}N_6O_{12}$	$[M+H]^+$	–	755.4185	755.4189	0.5
	$[M+Al-2H]^+$	–	779.3766	779.3781	1.9
	$[M+Fe-2H]^+$	–	808.3301	808.3318	2.1
DMA $C_{22}H_{36}N_4O_8$	$[M+H]^+$	–	485.2606	485.2605	-0.2
	$[M+Al-2H]^+$	–	509.2186	509.2191	1.0

MALDI+ Method Validation for the Detection of Cop	
**Validation Parameter**	**Results**
Linearity – Pearson's r	0.9999
LOD [ng/mL]	4.0
LOQ [ng/mL]	12.0
Precision 1 (250 ng/mL; expressed as relative standard deviation in %)	25
Precision 2 (500 ng/mL; expressed as relative standard deviation in %)	11
Precision 3 (2500 ng/mL; expressed as relative standard deviation in %)	5
Trueness 1 (250 ng/mL; expressed as recovery of Cop in %)	109
Trueness 2 (500 ng/mL; expressed as recovery of Cop in %)	97
Trueness 3 (2500 ng/mL; expressed as recovery of Cop in %)	99

Table S3. Overview of the proteins involved in the SIA of *Lp*, *Sa*, and *Af*. Proteins highlighted in green are enzymes responsible for the biosynthesis of extracellular and intracellular siderophores. Proteins highlighted in blue are transcription factors participating in the SIA, including zinc-inducible (^{Zn}) AtrR, AcuM, and SreA. The BLAST® software with BLASTP or BLASTX algorithm was used for the comparison of the protein similarity. *C*: cover [%], *E*: *E*-value, *I*: identity [%], *: pseudogenes in the draft genome sequence of *Sa*, **: old locus tag.

**Overview of the proteins involved in the SIA of *Lp***													
**Locus tag and the corresponding protein**				**Interspecies comparison of homology**									
***Lp* (JHH-5617)**	***Sa* (IHEM 14462)**	***Af* (Af293)**	**Protein**	***Lp* vs. *Af***			***Sa* vs. *Af***			***Sa* vs. *Lp***			
				***C* [%]**	***E***	***I* [%]**	***C* [%]**	***E***	***I* [%]**	***C* [%]**	***E***	***I* [%]**	
jhhlp_003063	SAPIO_CDS9033	AFUA_2G07680	SidA	85	10^{-162}	51	79	10^{-145}	49	98	0	72	
jhhlp_003062	SAPIO_CDS9032	AFUA_1G17200	SidC	96	0	26	96	0	27	100	0	67	
jhhlp_001563	SAPIO_CDS2806*	AFUA_3G03420	SidD	89	0	45	87	0	45	99	0	82	
jhhlp_001566	SAPIO_CDS2803*	AFUA_3G03400	SidF	92	10^{-139}	49	91	10^{-131}	46	96	0	87	
–	–	AFUA_3G03650	SidG	–	–	–	–	–	–	–	–	–	
jhhlp_005394	SAPIO_CDS2272*	AFUA_3G03410	SidH	100	10^{-101}	53	98	10^{-99}	53	99	10^{-166}	78	
jhhlp_001564	SAPIO_CDS2805	AFUA_1G17190	SidI	97	0	60	97	0	60	100	0	89	
jhhlp_001573	SAPIO_CDS2796**<br>SAPIO_CDS10921	AFUA_1G04450	SidL	90	10^{-133}	46	92	10^{-136}	46	100	0	80	
jhhlp_002472	SAPIO_CDS9713	AFUA_2G02690	$AtrR^{Zn}$	97	0	64	97	0	63	99	0	91	
jhhlp_005646	SAPIO_CDS0915	AFUA_2G12330	$AcuM^{Zn}$	56	10^{-112}	53	94	10^{-114}	43	76	0	78	
jhhlp_003678	SAPIO_CDS7310	AFUA_5G11260	$SreA^{Zn}$	42	10^{-55}	49	41	10^{-53}	46	92	0	81	
jhhlp_002449	SAPIO_CDS9738*	AFUA_5G03920	HapX	81	10^{-40}	33	36	10^{-20}	32	99	0	71	

Table S4. Overview of the *b*-ion series of Scedocyclin A and Scedocyclin B revealed upon the *de novo* sequencing of their corresponding CID mass spectra using CycloBranch. Green highlights *b*-ions with the same *m/z* values for both scedocyclins, whereas blue denotes *b*-ions differing in a methylene group (14.0157 Da). Peaks were selected with a minimum of 0.5% and 2% relative intensity for Scedocyclin A and Scedocyclin B, respectively.

Overview of the exact CID tandem mass spectrometry of Scedocyclin A and B										
Ion form	Scedocyclin A					Scedocyclin B				
	Composition	Calculated *m/z*	Measured *m/z*	Error [ppm]	Inten-sity [%]	Composition	Calcu-lated *m/z*	Measured *m/z*	Error [ppm]	Inten-sity [%]
$[M+H]^+$	$[C_{41}H_{71}N_9O_9+H]^+$	834.5448	834.5448	0.0	100	$[C_{40}H_{69}N_9O_9+H]^+$	820.5291	820.5291	0.0	26.1
b_8	Loss of Gly	777.5233	777.5265	4.2	0.7	–	–	–	–	–
b_8	Loss of Pro	737.4920	737.4950	4.1	1.0	–	–	–	–	–
b_8	Loss of Lxx	721.4607	721.4636	4.1	76.7	Loss of Lxx	707.4450	707.4449	-0.2	51.8
b_7	Loss of Abu, Gly	692.4705	692.4733	4.0	11.8	Loss of Abu, Gly	678.4549	678.4553	0.6	6.1
b_7	Loss of Abu, Abu	664.4392	664.4418	3.9	38.9	Loss of Abu, Abu	650.4236	650.4233	-0.5	31.5
b_7	Loss of Lxx, Pro	624.4079	624.4102	3.6	36.7	Loss of Val, Pro	624.4079	624.4078	-0.2	21.8
b_6	Pro, Lxx, Lxx, Pro, Lxx, Abu	619.4178	619.4200	3.5	25.4	Pro, Lxx, Lxx, Pro, Val, Abu	605.4021	605.4018	-0.5	20.8
b_7	Loss of Lxx, Lxx	608.3766	608.3788	3.6	10.6	Loss of Lxx, Lxx	594.3610	594.3612	0.4	8.2
b_6	Lxx, Lxx, Pro, Lxx, Abu, Abu	607.4178	607.4196	3.1	6.3	Lxx, Lxx, Pro, Val, Abu, Abu	593.4021	593.4020	-0.3	5.6
b_6	Lxx, Pro, Lxx, Abu, Abu, Gly	551.3552	551.3569	3.2	31.4	Lxx, Pro, Val, Abu, Abu, Gly	537.3395	537.3394	-0.3	30.9
b_6	Abu, Abu, Gly, C_3H_7NO, Pro, Lxx	511.3239	511.3254	2.9	57.7	Abu, Abu, Gly, C_3H_7NO, Pro, Lxx	511.3239	511.3236	-0.4	55.6
b_5	Lxx, Lxx, Pro, Lxx, Abu	522.3650	522.3666	3.1	12.7	Lxx, Lxx, Pro, Val, Abu	508.3494	508.3491	-0.4	6.5
b_5	Lxx, Pro, Lxx, Abu, Abu	494.3337	494.3351	2.7	53.4	Lxx, Pro, Val, Abu, Abu	480.3181	480.3178	-0.5	56.9
b_5	Gly, C_3H_7NO, Pro, Lxx, Lxx	454.3024	454.3036	2.7	22.4	Gly, C_3H_7NO, Pro, Lxx, Lxx	454.3024	454.3022	-0.4	27.4
b_5	Pro, Lxx, Abu, Abu, Gly	438.2711	438.2723	2.7	4.1	Pro, Val, Abu, Abu, Gly	424.2555	424.2554	-0.1	5.0
b_4	Lxx, Lxx, Pro, Lxx	437.3122	437.3134	2.8	2.3	Lxx, Lxx, Pro, Val	423.2966	423.2966	0.1	2.7
b_5	Abu, Abu, Gly, C_3H_7NO, Pro	398.2398	398.2408	2.4	8.0	Abu, Abu, Gly, C_3H_7NO, Pro	398.2398	398.2397	-0.4	13.3
b_4	Lxx, Pro, Lxx, Abu	409.2809	409.2819	2.4	22.5	Lxx, Pro, Val, Abu	395.2653	395.2650	-0.8	6.6
b_4	Pro, Lxx, Abu, Abu	381.2496	381.2505	2.2	22.1	Pro, Val, Abu, Abu	367.2340	367.2338	-0.4	35.5
b_4	Gly, C_3H_7NO, Pro, Lxx	341.2183	341.2191	2.1	7.2	Gly, C_3H_7NO, Pro, Lxx	341.2183	341.2182	-0.5	16.3
b_3	Lxx, Pro, Lxx	324.2282	324.2288	1.9	10.5	Lxx, Pro, Val	310.2125	310.2124	-0.5	25.4
b_2	Pro, Lxx	211.1441	211.1443	1.1	0.9	Pro, Val	197.1285	197.1284	-0.5	3.4
